# Bidirectional associations between smart device use and body mass index among children aged 3 to 5 years: a longitudinal study

**DOI:** 10.1186/s12966-026-01883-3

**Published:** 2026-02-04

**Authors:** Pairote Chakranon, Jian-Pei Huang, Heng-Kien Au, Hawjeng Chiou, Chen-Li Lin, Yi-Yung Chen, Shih-Peng Mao, Pilyoung Kim, Hsueh-Wen Hsu, Yi-Hua Chen

**Affiliations:** 1 School of Public Health, College of Public Health, Taipei Medical University, New Taipei, Taiwan; 2 Department of Obstetrics and Gynecology, Mackay Memorial Hospital, Taipei, Taiwan; 3 Department of Obstetrics and Gynecology, Taipei Medical University Hospital, Taipei, Taiwan; 4 Department of Obstetrics and Gynecology, School of Medicine, College of Medicine, Taipei Medical University, Taipei, Taiwan; 5 Department of Business Administration, College of Management, National Taiwan Normal University, Taipei, Taiwan; 6 Department of Obstetrics & Gynecology, Taipei City Hospital, Heping Fuyou Branch, Taipei, Taiwan; 7 Department of Obstetrics and Gynecology, Taipei Medical University-Shuang Ho Hospital, Ministry of Health and Welfare, New Taipei, Taiwan; 8 Department of Psychology, College of Arts, Humanities and Social Sciences, University of Denver, Denver, CO USA; 9 Department of Psychology, Ewha Womans University, Seoul, South Korea; 10 Master Program in Applied Epidemiology, College of Public Health, Taipei Medical University, Taipei, Taiwan; 11 Neuroscience Research Center, Taipei Medical University, Taipei, Taiwan; 12 Research Center of Health Equity, College of Public Health, Taipei Medical University, New Taipei, Taiwan

**Keywords:** Smart device use, Body mass index, Preschooler, Mother-child interactions

## Abstract

**Background:**

The increase in smart device use, including smartphones and tablets, among young children has raised concerns about its impact on health, particularly on body mass index (BMI). However, the bidirectional associations between smart device use and BMI in preschoolers remain unclear. This study examined the longitudinal associations, considering the moderating effects of mother-child interactions and child sex.

**Methods:**

Data were obtained from the Longitudinal Examination Across Prenatal and Postpartum Health in Taiwan, a cohort study conducted in Taipei, Taiwan. In total, 590 preschoolers were assessed at ages 3, 4, and 5 years. Smart device use, BMI z-scores, and mother-child interaction quality were evaluated using validated parent-reported questionnaires. The random-intercept cross-lagged panel model was used to investigate bidirectional associations, adjusting for stable confounders. Multiple-group models examined the moderating effects of mother-child interactions and child sex. Model estimates were reported as standardized coefficients.

**Results:**

Higher BMI z-scores at age 4 years were linked to increased device use at age 5 years (β = 0.36; 95% CI, 0.05–0.67). Multiple-group models revealed that among dyads with lower mother-child interactions, higher device use at age 3 years was associated with higher BMI at age 4 years (β = 0.40; 95% CI, 0.07 to 0.72), which was subsequently linked to greater device use at age 5 years (β = 0.50; 95% CI, 0.10 to 0.90). Additionally, higher device use at age 4 years was associated with higher BMI at age 5 years (β = 0.65; 95% CI, 0.31 to 1.00). A similar bidirectional pattern was observed among boys, while no significant cross-lagged associations were found among girls. In contrast, high-quality mother-child interactions revealed higher device use at age 4 years was associated with lower BMI at age 5 years, suggesting a protective role against prolonged device use and subsequent BMI increases.

**Conclusions:**

Our study indicates bidirectional associations between smart device use and BMI among preschoolers, emphasizing the protective role of high-quality mother-child interactions. Interventions should focus on enhancing parent-child relationships, limiting device use, and promoting active engagement. Future studies should investigate the effect of media content and children’s self-regulation on these associations.

**Supplementary Information:**

The online version contains supplementary material available at 10.1186/s12966-026-01883-3.

## Background

Screen time is regarded as a global concern, particularly when introduced at an early age [1, 2]. Excessive exposure to screens during early childhood is associated with negative developmental outcomes, including delays in language acquisition, social skills, and cognitive growth [3–8]. It is also associated with adverse physical health outcomes, including an increased body mass index (BMI) [9–12], decreased sleep duration [13, 14], and an increased risk of musculoskeletal problems [15, 16]. Among these outcomes, elevated BMI z-scores are a concern because they reflect excessive weight relative to age- and sex-specific growth norms and serve as a key indicator of childhood obesity [17], particularly among preschool-aged children [18]. Evidence suggests that elevated BMI z-scores during early childhood are associated with higher risks of obesity and related health problems later in life [19–22]. Although research on early childhood screen use is growing, only a few studies have examined the unique characteristics of interactive smart devices, compared to traditional screen forms like television [9–11]. This shift in screen use underscores the need to determine how smart devices contribute to the risk of childhood obesity [23].

Smart devices, including smartphones and tablets [24], provide interactive, personalized, and hands-on experiences that deliver stronger auditory and visual stimulation, whereas television primarily involves passive viewing. Given the widespread use of smart devices in today’s digital age, they may have distinct and profound effects on multiple aspects of early childhood development [2, 25, 26]. For instance, interactive applications from smartphones or tablets can support early literacy and vocabulary when parents co-engage with children [25], whereas excessive or unsupervised use has been linked to reduced parent-child interactions and increased sedentary behaviors [25, 26]. Recent longitudinal evidence further shows that early tablet use is associated with greater emotional dysregulation [8].

Many studies have reported a positive relationship between screen time and child BMI, a relationship likely attributable to sedentary lifestyle and unhealthy eating habits associated with prolonged screen use [9–12]. Few studies have investigated how BMI itself may affect patterns of screen time, particularly with interactive smart devices. Children with higher BMI often have fewer opportunities for vigorous physical activity, increasing their reliance on sedentary behaviors such as device use [27, 28]. In addition to BMI itself, several correlates that commonly accompany higher BMI, such as sleep difficulties and emerging self-regulatory challenges, may also relate to children’s screen use [29, 30]. However, these associations remain tentative, particularly in preschool-aged children, and require further investigation. Together, these findings highlight the plausibility of reciprocal associations between BMI and smart device use, particularly during early childhood, a critical period for establishing lifelong habits. Recognizing this bidirectional relationship could allow interventions to address both smart device use and BMI, as well as related factors such as sleep, fitness, and self-regulation, thereby enhancing effectiveness, supporting healthier development, and reducing long-term health risks.

Maternal sensitivity and responsiveness, key components of mother-child interactions that are crucial for healthy development, may mitigate the adverse effects of excessive screen time [31, 32]. In addition, boys and girls may display distinct screen use patterns, which can differently influence their BMI outcomes [11, 33]. Despite growing evidence linking screen use to childhood adiposity, few longitudinal studies have concurrently examined how BMI may also influence subsequent smart device use, particularly among preschool-aged children. To address this gap, in the present study, we examined the reciprocal relationship between smart device use and BMI in preschoolers aged 3 to 5 years, using a longitudinal design with the random-intercept cross-lagged panel model (RI-CLPM), while accounting for the moderating roles of mother-child interactions and child sex [11, 33–35]. In addition, we examined time-invariant parental, family, and child characteristics as covariates predicting between-person differences in children’s smart device use and BMI, to provide contextual insight into stable individual- and family-level factors underlying these longitudinal associations. By incorporating these moderators along with relevant covariates, we aimed to adequately examine the dynamic interplay among smart device use, BMI, and the family context, evidence that could inform early interventions to prevent obesity and promote healthier behaviors for long-term health benefits.

## Methods

### Study design and data collection

Parents and children were recruited from the Longitudinal Examination Across Prenatal and Postpartum Health in Taiwan (LEAPP-HIT), an ongoing prospective study initiated in 2011 in metropolitan Taipei. Pregnant women and their partners were consecutively approached and invited to participate during their early prenatal visits at five selected hospitals, where baseline data were collected. Participants were subsequently followed up through postal surveys up to the sixth postpartum year. Telephone reminders were used to improve the response rate. Additional details regarding the LEAPP-HIT project are available elsewhere [36].

We included Mandarin-speaking women aged ≥ 20 years who were in early pregnancy (< 16 weeks), intended to carry to full term, and had partners willing to participate. We specifically examined data on smart device use and BMI collected during the 3-, 4-, and 5-year postpartum follow-up assessments. Demographic characteristics of the children and their parents, as well as variables related to maternal lifestyle and psychosocial factors, are presented in Table 1. Written informed consent was obtained from all participants, and the study protocol was approved by the institutional review boards of all participating hospitals.

**Table 1:** Characteristics of children and parents (*N* = 590)

Characteristic	Value ^a^
Maternal age, n (%)	
< 35	361 (61.82)
≥ 35	223 (38.18)
Maternal BMI, ^b^ mean (SD)	22.60 (3.73)
Maternal depression, ^c^ n (%)	
Low	458 (84.35)
High	85 (15.65)
Mother-child interactions, ^d^ n (%)	
Lower	339 (65.83)
Higher	176 (34.17)
Children’s outdoor activities, n (%)	
≥ 1 time/day	52 (9.61)
Once a day	97 (17.93)
2–4 times/week	284 (52.50)
2–4 times/month	76 (14.05)
Once a month	18 (3.33)
Never	14 (2.59)
Paternal BMI, ^b^ mean (SD)	25.41 (3.62)
Parental educational level, n (%)	
Both parents with a graduate school degree or higher	324 (55.29)
Either parent with a graduate school degree or higher	166 (28.33)
Both parents with a college degree or lower	96 (16.38)
Family monthly income, ^e^ n (%)	
≤ NTD100 000	347 (59.62)
> NTD100 000	235 (40.38)
Parity, n (%)	
Primiparous	355 (60.17)
Multiparous	235 (39.83)
Gestational age (weeks), n (%)	
< 37	45 (7.64)
≥ 37	544 (92.36)
Child sex, n (%)	
Boy	299 (50.68)
Girl	291 (49.32)
Child’s smart device use (hours/day), mean (SD)	
At age 3	0.57 (0.78)
At age 4	0.55 (0.89)
At age 5	0.52 (0.77)
Child’s BMI (*z*-scores), mean (SD)	
At age 3	0.13 (1.07)
At age 4	0.14 (1.16)
At age 5	0.09 (1.11)

*Abbreviations*: *BMI* body mass index, *SD* standard deviation, *NTD* New Taiwan dollar

^a^ The total for each variable may vary due to missing values

^b^ Maternal and paternal BMI were evaluated when the child was 3 years old

^c^ Maternal depression at the child’s age of 3 years was evaluated using the 10-item Edinburgh Postnatal Depression Scale, with a cutoff score of 13 indicating a high level of depression

^d^ Mother-child interactions at the child’s age of 3 years were measured using the Brigance Parent-Child Interactions Scale and categorized into lower and higher levels on the basis of the third quartile. Because of tied scores, the proportions do not exactly align with the intended 25% versus 75% division

^e^ Average exchange rate in 2023: US$1.00 = NTD30.03

### Sample

The initial sample included 866 eligible parent-child dyads with singleton births after December 2016, when information on children’s smart device use was first collected in the cohort. Following the approach used in a similar pediatric RI-CLPM study [8] with greater methodological rigor, dyads were included if they had at least one time point with concurrent information on both smart device use and BMI z-scores during the 3-, 4-, or 5-year follow-ups, with assessments conducted through September 2024. Dyads with no concurrent measures across any wave (e.g., missing both variables entirely or having only one variable available at any time point; *n* = 276) were excluded, resulting in a final analytic sample of 590 participants. Comparisons of baseline characteristics between included and excluded participants are presented in Supplemental Table S1, demonstrating no significant differences between the two groups.

### Measures

#### Child smart device use

In this study, smart device use specifically referred to smartphones and tablets [24]. Mothers reported their children’s smart device use at the ages of 3, 4, and 5 years for typical weekdays and weekends by responding to questions such as “How long does your child usually spend on smart devices (e.g., smartphones and tablets) in total?” with responses provided in hours and minutes per day. The daily mean time spent on smart devices was calculated in units of hours per day as a continuous variable, using the weighted formula: [(time on weekdays × 5) + (time on weekends × 2)]/7 [37]. This approach was consistent with that used in previous studies [4, 37]. Maternal reports of children’s screen use are widely accepted in early-childhood research, with a systematic review showing parental reporting as the most common measure for screen time in children aged 0–6 years [38]. Although adequate validation data in preschoolers are lacking, evidence from young adults indicates that self-reported digital media time shows moderate correspondence with device-log measures (*r* =.51) [39].

#### Child BMI z-scores

Mothers provided information on their children’s height and weight at the ages of 3, 4, and 5 years, based on measurements recorded in the *Children’s Health Handbook*, which is maintained by health professionals during routine well-child clinic checkups. BMI *z*-scores, adjusted for age and sex, were calculated as per the World Health Organization’s standards, which are recommended by national health authorities as appropriate for the Taiwanese pediatric population under 5 years of age [40].

### Moderating variables

#### Mother-child interactions

Mother-child interactions at the age of 3 years were evaluated using the Brigance Parent-Child Interactions Scale [41], an 18-item tool with established reliability and validity [42, 43]. In this analysis, total scores, with higher values indicating greater interaction levels, were converted into a binary variable. The upper 25% of scores were classified as a higher interaction level, whereas the lower 75% were classified as a lower interaction level (quartile 3) [44]. We applied the 75th percentile as the cutoff; however, because this value corresponded to a tied score, the sample was not split into exactly 25% and 75%, although the categorization was still based on the 75th-percentile threshold (Table 1).

#### Child sex

Child sex was collected via maternal report at the 1-month postpartum survey, based on the sex recorded at birth in the *Children’s Health Booklet* by healthcare providers.

#### Covariates

In accordance with previous studies [6, 7, 34, 45–48], we included the following covariates at the baseline to examine time-invariant factors affecting smart device use and BMI. During early pregnancy, parents completed a baseline survey that collected information on their sociodemographic factors, including age, educational level, parity, and family monthly income. Maternal age was categorized using a cutoff of 35 years to define an advanced maternal age [49]. Gestational age was classified as term (≥ 37 weeks) or preterm. At the age of 3 years, the following covariates were assessed and treated as baseline measures in the analyses. Maternal depression was assessed using the 10-item Edinburgh Postnatal Depression Scale [50], with scores of 13 or greater indicating higher levels of depression [51]. In addition, mothers reported the frequency of outdoor activities with their children responding to the item: “Taking your child out for a walk or to the yard, park, or playground.” Responses were recorded on a 6-point Likert scale ranging from 1 (never) to 6 (≥ 1 time/day). This variable was treated as continuous, with higher scores indicating a greater frequency of outdoor activities. Parental BMI was calculated from self-reported height and weight and treated as continuous variables in the analyses.

### Statistical analyses

Descriptive statistics were used to summarize participant characteristics. We employed the RI-CLPM to examine the longitudinal association between smart device use and BMI *z*-scores in children aged 3 to 5 years [52]. This model is regarded as an improvement on the traditional cross-lagged panel model by decomposing variance into between-person and within-person components. Between-person components capture stable individual differences through random intercepts for smart device use and BMI *z*-scores, whereas within-person components identify time-varying fluctuations, isolating how deviations in one variable relate to changes in another. This method sets each participant as their own baseline control, adjusting for time-stable between-person differences (e.g., socioeconomic status).

Our analysis involved three steps. First, we implemented the basic RI-CLPM by using observed BMI *z*-scores and smart device use at each time point (Supplementary Appendix). Second, following an approach proposed by Mulder and Hamaker [53], we extended the RI-CLPM using multiple-group models to examine whether structural paths differed across mother-child interaction groups (higher vs. lower) and across child sex (boys vs. girls). We conducted chi-square difference tests to compare models with unconstrained parameters (where coefficients were freely estimated) against those with constrained parameters (where coefficients were fixed across groups). Significant outcomes from these tests indicated the presence of moderation effects, demonstrating differential impacts of these moderators. Finally, we included baseline covariates as predictors of random intercepts (between-person components), with all covariates simultaneously specified within the structural model to examine their associations with children’s smart device use and BMI [53].

Model fit for the RI-CLPMs was evaluated on the basis of Hu and Bentler’s criteria [54]. Adequate fit was indicated by a comparative fit index (CFI) of > 0.95, a root mean square error of approximation (RMSEA) of < 0.06, and a standardized root mean square residual (SRMSR) of < 0.06. Notably, the value of χ^2^ was small and did not reach statistical significance (*p* >.05). All statistical analyses were conducted using Mplus version 8.11 [55]. All models were reported using standardized estimates (β), with statistical significance determined at *p* <.05 (two-tailed) with 95% confidence intervals (CIs).

All models were estimated using maximum likelihood estimations with robust standard errors to account for data nonnormality. Full-information maximum likelihood was used to handle missing data. Little’s missing completely at random test [56] indicated that missing data for key variables, including smart device use and BMI z-scores, were missing completely at random (χ^2^ = 175.26, *p* =.70). This finding suggests that the pattern of missing data and participant attrition was unrelated to children’s smart device use or BMI z-scores.

## Results

### Descriptive statistics

Characteristics of the children and their parents are summarized in Table 1. Of a total of 590 children, 50.68% were boys and 38.18% had mothers aged ≥ 35 years. In addition, 57.46% of mother-child interactions were classified as lower level. The mean (SD) ages of children at the 3-, 4-, and 5-year assessments were 3.60 (± 0.20), 4.50 (± 0.30), and 5.40 (± 0.30) years, respectively. The average daily smart device use was 0.57 (± 0.78), 0.55 (± 0.89), and 0.52 (± 0.77) hours at the ages of 3, 4, and 5 years, respectively. The mean (SD) BMI *z*-scores were 0.13 (± 1.07), 0.14 (± 1.16), and 0.09 (± 1.11) at the ages of 3, 4, and 5 years, respectively.

### Bidirectional associations between smart device use and BMI z-scores

**Fig. 1:**
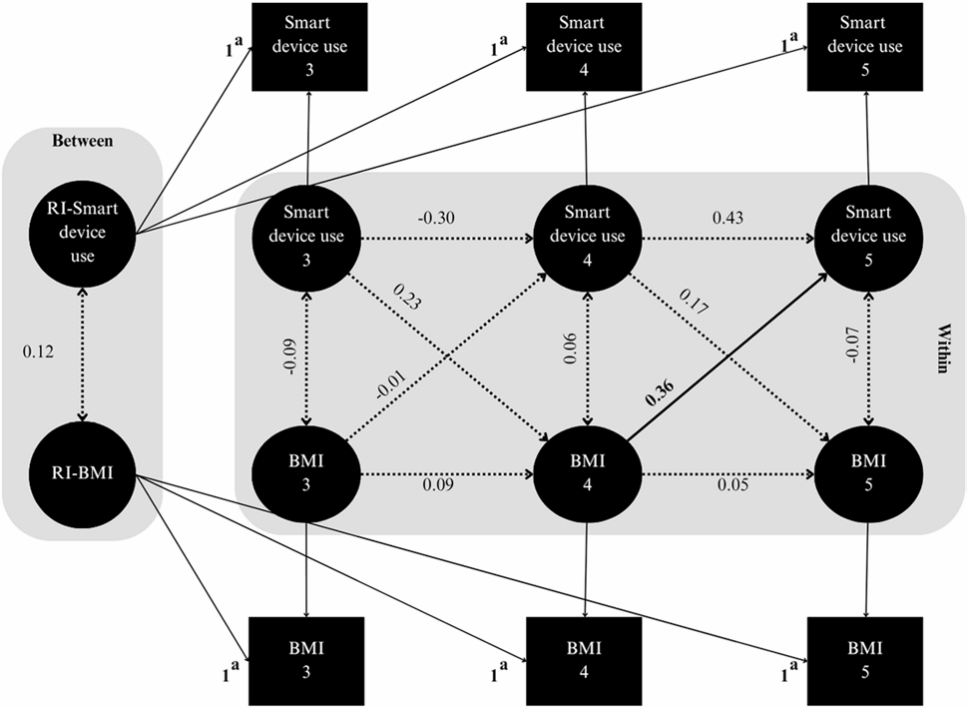
Random-intercept cross-lagged panel model examining between-person (random intercept) and within-person (autoregressive and cross-lagged) associations between smart device use and BMI in preschoolers. Standardized estimates are presented; solid lines and bold values indicate significant paths. RI: random intercept. ^a^ Factor loadings were constrained to 1.00 to isolate between-person differences in smart device use and BMI

The basic RI-CLPM demonstrated excellent fit indices (Fig. 1, Supplemental Table S2). Significant variances in random intercepts were identified for both smart device use (σ^2^ = 0.39, 95% CI = 0.18 to 0.60) and BMI *z*-scores (σ^2^ = 0.67, 95% CI = 0.46 to 0.88), indicating stable individual differences in these measures and supporting the inclusion of a random intercept in the model. In the within-person cross-lagged effect, we observed a significant positive association between BMI *z*-scores at the age of 4 years and higher smart device use at the age of 5 years (β = 0.36, 95% CI = 0.05 to 0.67).

### Moderation effects

Model fit indices and results from chi-square difference tests comparing unconstrained and constrained models within the RI-CLPM framework are presented in Supplemental Table S3. A significant chi-square difference was observed, indicating that mother-child interactions and child sex significantly moderated the longitudinal association between smart device use and BMI *z*-scores. Both moderation models demonstrated a good fit to the data.

**Fig. 2:**
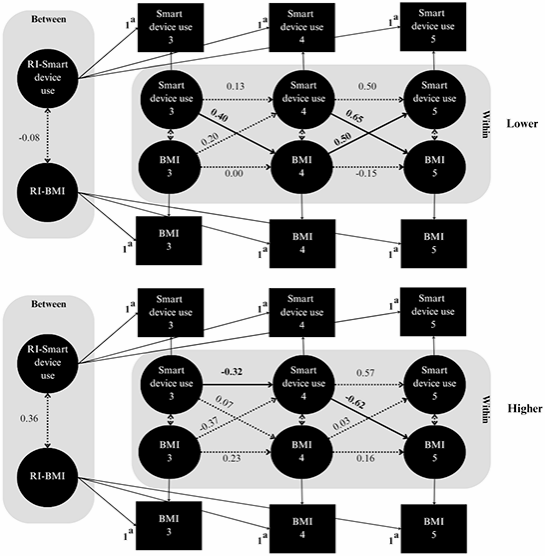
Random-intercept cross-lagged panel model examining between-person (random intercept) and within-person (autoregressive and cross-lagged) associations between smart device use and BMI in preschoolers, stratified by mother-child interactions. A multigroup analysis was used to compare lower and higher interaction levels. Standardized estimates are presented; solid lines and bold values indicate significant paths. RI: random intercept. ^a^ Factor loadings were constrained to 1 to isolate between-person differences in smart device use and BMI

Figure 2 and Table 2 present the results of the multiple-group RI-CLPM analysis, which examined the moderating effects of mother-child interaction levels. Among children in the lower interaction group, significant cross-lagged associations were observed. Elevated smart device use at the age of 3 years was associated with increased BMI *z*-scores at the age of 4 years (β = 0.40, 95% CI = 0.07 to 0.72), which in turn was linked to increased smart device use at the age of 5 years (β = 0.50, 95% CI = 0.10 to 0.90). In addition, higher smart device use at the age of 4 years was associated with increased BMI at the age of 5 years (β = 0.65, 95% CI = 0.31 to 1.00). In contrast, in the higher interaction group, increased smart device use at the age of 4 years was associated with decreased BMI *z*-scores at the age of 5 years (β = −0.62, 95% CI = − 1.02 to − 0.21). An autoregressive effect was also observed, indicating a reduction in smart device use from the age of 3 to 4 years (β = −0.32, 95% CI = − 0.62 to − 0.03).

**Table 2:** Directional associations of autoregressive and cross-lagged coefficients between children’s smart device use and BMI, stratified by mother-child interactions^a^

Association	Standardized estimate (95% CI)	
	Lower interaction	Higher interaction
Cross-lagged effects		
* Ages 3 to 4*		
Smart device use at age 3 → BMI at age 4	**0.40 (0.07**,** 0.72)**	0.07 (− 0.23, 0.36)
BMI at age 3 → Smart device use at age 4	0.20 (− 0.05, 0.44)	−0.37 (− 0.92, 0.18)
* Ages 4 to 5*		
Smart device use at age 4 → BMI at age 5	**0.65 (0.31**,** 1.00)**	**−0.62 (− 1.02**,** − 0.21)**
BMI at age 4 → Smart device use at age 5	**0.50 (0.10**,** 0.90)**	0.03 (− 0.28, 0.34)
Autoregressive effects		
* Ages 3 to 4*		
Smart device use at age 3 → Smart device use at age 4	0.13 (− 0.20, 0.46)	**−0.32 (− 0.62**,** − 0.03)**
BMI at age 3 → BMI at age 4	0.00 (− 0.42, 0.42)	0.23 (− 0.44, 0.89)
* Ages 4 to 5*		
Smart device use at age 4 → Smart device use at age 5	0.50 (− 0.02, 1.02)	0.57 (− 0.20, 1.34)
BMI at age 4 → BMI at age 5	−0.15 (− 0.58, 0.28)	0.16 (− 0.48, 0.80)
Within-time covariances		
Smart device use at age 3 ↔ BMI at age 3	0.10 (− 0.26, 0.46)	−0.27 (− 0.66, 0.12)
Smart device use at age 4 ↔ BMI at age 4	0.13 (− 0.10, 0.37)	0.04 (− 0.24, 0.32)
Smart device use at age 5 ↔ BMI at age 5	0.14 (− 0.28, 0.56)	−0.22 (− 0.96, 0.52)
Between-person covariance		
B-Smart device ↔ B-BMI	−0.08 (− 0.62, 0.45)	0.36 (− 0.04, 0.76)

Bold values indicate standardized estimates where 95% CIs do not include zero

^a^ Mother-child interactions were evaluated using the Brigance Parent-Child Interactions Scale when the child was 3 years old

**Fig. 3:**
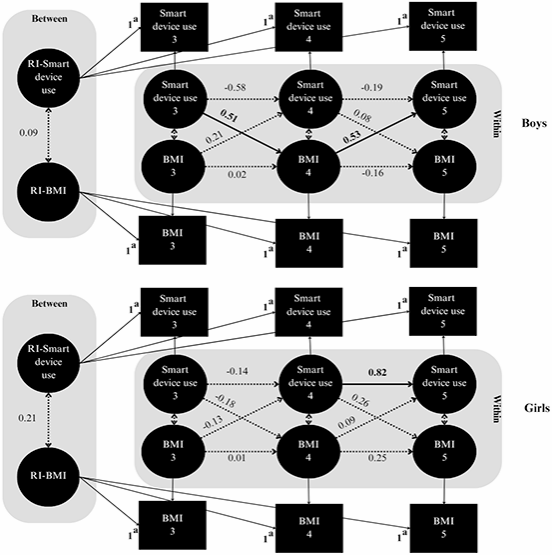
Random-intercept cross-lagged panel model examining between-person (random intercept) and within-person (autoregressive and cross-lagged) associations between smart device use and BMI in preschoolers, stratified by child sex. A multigroup analysis was used to compare boys and girls. Standardized estimates are presented; solid lines and bold values indicate significant paths. RI: random intercept. ^a^ Factor loadings were constrained to 1 to isolate between-person differences in smart device use and BMI

Figure 3 and Table 3 illustrate the moderating effect of child sex. Among boys, increased smart device use at the age of 3 years was associated with increased BMI *z*-scores at the age of 4 years (β = 0.51, 95% CI = 0.17 to 0.86), which was subsequently correlated with increased smart device use at the age of 5 years (β = 0.53, 95% CI = 0.20 to 0.86). In contrast, no cross-lagged associations were observed for girls, except for a significant positive relationship between smart device use at the age of 4 years and smart device use at the age of 5 years (β = 0.82, 95% CI = 0.60 to 1.04).

**Table 3:** Directional associations of autoregressive and cross-lagged coefficients between children’s smart device use and the body mass index (BMI), stratified by child sex^a^

Association	Standardized estimate (95% CI)	
	Boys	Girls
Cross-lagged effects		
* Ages 3 to 4*		
Smart device use at age 3 → BMI at age 4	**0.51 (0.17**,** 0.86)**	−0.18 (− 0.58, 0.23)
BMI at age 3 → Smart device use at age 4	0.21 (− 0.32, 0.75)	−0.13 (− 0.41, 0.15)
* Ages 4 to 5*		
Smart device use at age 4 → BMI at age 5	0.08 (− 0.54, 0.70)	0.26 (− 0.18, 0.70)
BMI at age 4 → Smart device use at age 5	**0.53 (0.20**,** 0.86)**	0.09 (− 0.15, 0.33)
Autoregressive effects		
* Ages 3 to 4*		
Smart device use at age 3 → Smart device use at age 4	−0.58 (− 1.75, 0.59)	−0.14 (− 0.48, 0.19)
BMI at age 3 → BMI at age 4	0.02 (− 0.43, 0.47)	0.01 (− 0.75, 0.74)
* Ages 4 to 5*		
Smart device use at age 4 → Smart device use at age 5	−0.19 (− 0.86, 0.48)	**0.82 (0.60**,** 1.04)**
BMI at age 4 → BMI at age 5	−0.16 (− 0.70, 0.39)	0.25 (− 0.24, 0.73)
Within-time covariances		
Smart device use at age 3 ↔ BMI at age 3	0.07 (− 0.33, 0.46)	−0.28 (− 0.60, 0.03)
Smart device use at age 4 ↔ BMI at age 4	0.46 (− 1.20, 2.12)	−0.11 (− 0.42, 0.20)
Smart device use at age 5 ↔ BMI at age 5	−0.14 (− 0.50, 0.22)	0.09 (− 0.42, 0.60)
Between-person covariance		
B-Smart device ↔ B-BMI	0.09 (− 0.09, 0.27)	0.21 (− 0.11, 0.53)

Bold values indicate standardized estimates where 95% CIs do not include zero

^a^ *z*-scores for BMI-for-age were calculated in boys and girls using the World Health Organization’s reference data

### Predictors of smart device use and BMI z-scores

Table 4 presents a summary of the effects of time-invariant predictors on smart device use and BMI *z*-scores within the between-person components, demonstrating strong model fit indices. Increased smart device use was positively associated with increased maternal BMI (β = 0.14, 95% CI = 0.01 to 0.27) and paternal BMI (β = 0.15, 95% CI = 0.02 to 0.27). In contrast, increased family income (β = −0.19, 95% CI = − 0.30 to − 0.08) and maternal engagement in outdoor activities (β = −0.14, 95% CI = − 0.27 to − 0.02) were associated with decreased smart device use. Child BMI *z*-scores were positively associated with both maternal BMI (β = 0.23, 95% CI = 0.12 to 0.34) and paternal BMI (β = 0.11, 95% CI = 0.01 to 0.22) but negatively associated with gestational age (β = −0.12, 95% CI = − 0.23 to − 0.02).

**Table 4:** Predictors of smart device use and BMI z-scores among preschoolers

Predictor	Standardized estimate (95% CI)	
	Smart device use	Child’s BMI
Maternal age	0.04 (− 0.08 to 0.17)	0.07 (− 0.05 to 0.18)
Maternal BMI	**0.14 (0.01 to 0.27)**	**0.23 (0.12 to 0.34)**
Maternal depression	0.05 (− 0.06 to 0.17)	−0.02 (− 0.13 to 0.09)
Children’s outdoor activities	**−0.14 (− 0.27 to − 0.02)**	0.06 (− 0.04 to 0.17)
Paternal BMI	**0.15 (0.02 to 0.27)**	**0.11 (0.01 to 0.22)**
Parental educational level	−0.07 (− 0.18 to 0.04)	0.08 (− 0.03 to 0.19)
Family monthly income	**−0.19 (− 0.3 to − 0.08)**	−0.02 (− 0.13 to 0.09)
Parity	0.09 (− 0.04 to 0.23)	0.06 (− 0.05 to 0.17)
Gestational age	−0.04 (− 0.14 to 0.06)	**−0.12 (− 0.23 to − 0.02)**
*R* ^2^	0.14	0.11
Fit indices		
χ^2^ (df), *p* value	38.98 (37), 0.382	
RMSEA	0.01	
CFI	0.99	
SRMR	0.04	

Bold values indicate standardized estimates where 95% CIs do not include zero.

*Abbreviations*: *BMI *body mass index, *RMSEA* root mean square error of approximation, *CFI* Comparative fit index, *SRMR* standardized root mean square residual

## Discussion

To the best of our knowledge, this is the first study to examine the bidirectional relationship between smart device use and BMI among preschoolers aged 3 to 5 years using the RI-CLPM framework. Overall, we found evidence of reciprocal associations between smart device use and BMI, but these patterns differed by mother-child interactions and child sex. Bidirectional associations were primarily observed among children with lower mother-child interactions and among boys, whereas associations were weaker or absent among children with higher interactions and among girls. These findings highlight the potential importance of early family environments and child characteristics in shaping how smart device use and BMI are related over time.

Previous studies reported a positive association between screen time and child BMI [9–12]. For instance, a study of preschoolers aged 2–6 years found that higher television viewing was modestly associated with higher BMI z-scores [9]. Similarly, a longitudinal study that examined children under 5 years of age reported that greater screen viewing was linked to higher BMI [11]. Among older children (e.g., around age 11 years) and adults, increased BMI has been observed to be correlated with increased sedentary behaviors [27, 57]. Given these findings, we examined the moderating role of mother-child interactions in the bidirectional relationship between smart device use and BMI among preschoolers. By shifting the focus from traditional screens to interactive smart devices, we addressed an understudied dimension of this relationship. Our findings indicated that children with a low level of mother-child interactions exhibited a significant association between extensive smart device use and increased BMI. In addition, increased BMI was found to be correlated with increased smart device use over time. By contrast, higher levels of mother-child interactions appeared to buffer these effects, indicating a protective role of engaged parenting during early childhood.

Excessive smart device use may influence BMI through both biological and behavioral pathways. Biologically, short-wavelength blue light from electronic devices may suppress melatonin, disrupt circadian rhythms, and interfere with sleep [58]. Children are especially sensitive due to larger pupil sizes and greater lens transparency, resulting in higher retinal exposure [58]. Such disruption can contribute to delayed bedtimes and shorter sleep durations, both of which are well-established risk factors for increased BMI in childhood [23, 59, 60]. Behaviorally, increased screen time can displace physical activity and encourage unhealthy eating behaviors, such as frequent snacking during media use and heightened exposure to food advertising and eating cues, thereby elevating the risk of higher BMI [9–12].

Conversely, children with increased BMI may experience physical discomfort during physical activities, making sedentary behaviors such as screen use more appealing [61]. Several BMI-related factors may also contribute to this pattern. For example, an actigraphic study in children aged 5–6 years showed that those with excess adiposity tended to have shorter sleep durations, which may contribute to daytime tiredness and, in turn, reduce energy for active play and increase engagement in sedentary activities [29, 62]. In addition, increased BMI has been associated with impaired development of the prefrontal cortex, a brain region essential for executive functioning [30]. This impairment could hinder self-regulation and impulse control, making it more challenging for children to limit their engagement with devices [5].

The quality of mother-child interactions plays a pivotal role in the bidirectional relationship between smart device use and BMI. High-quality interactions support healthier behaviors by establishing screen-time boundaries and encouraging physical activity, thereby offsetting potential increases in BMI [32, 63–66]. Within such interactions, higher device use may coexist with greater physical activity, helping to prevent BMI from rising. These interactions also foster the development of self-regulatory skills, enabling children to manage behaviors such as excessive screen use [32, 67]. In contrast, lower levels of mother-child interactions may hinder the development of self-regulatory abilities and fail to promote a healthy lifestyle [68]. This lack of guidance may make children more susceptible to sedentary behaviors and the engaging stimuli of smart devices, increasing the likelihood of prolonged media use [5, 68, 69]. It is worth noting that because the third quartile was used as the cutoff for interaction levels, the lower 75% represents the majority of children with typical mother-child interactions, whereas the upper 25% reflects substantially higher-than-normal levels that may be necessary to buffer the impact of greater device use on BMI.

Our findings revealed sex-specific effects, particularly among boys. Smart device use at the age of 3 years was associated with increased BMI at the age of 4 years, which was subsequently correlated with increased smart device use at the age of 5 years. This pattern aligns with prior evidence that indicates an association between screen exposure and adiposity among young boys. For example, a longitudinal cohort of children followed from ages 2–3 to 3–5 years found that greater screen viewing was linked to higher BMI and greater skinfold thickness in boys but not girls [11]. Similarly, among older children aged 9–16 years, screen time showed stronger associations with being overweight and obese than with physical activity, especially among boys [33]. These findings support the possibility that boys may be more sensitive to screen-related behavioral patterns that contribute to higher BMI. For instance, boys may spend more time on screens, often engaging with highly stimulating digital content, such as video games, which can drive greater demands for screen use. In contrast, girls may be more likely to engage with different types of media than boys, although findings vary across studies [70, 71]. These screen-based behaviors may replace physical activity, contributing to gradual weight gain. Furthermore, boys are more susceptible to higher BMI and encounter greater difficulties with self-regulation compared to girls, making them particularly vulnerable to prolonged screen use [5, 18, 71, 72]. 

Other factors likely contribute to the relationship between preschoolers’ smart device use and BMI. Specifically, we found that both maternal and paternal BMI were associated with increases in children’s smart device use and BMI. This finding indicates the effects of familial and environmental factors on screen behaviors and child BMI. Consistent with findings of previous studies [34, 46, 48], we suggested that increased parental BMI contributes to an obesogenic environment, in which parents may engage less in physical activities with their children, leading to increased smart device use and fewer opportunities for developing active habits. In contrast, maternal behaviors, such as taking children to the park, and higher family income were associated with decreased smart device use, consistent with results of a previous study [6]. In addition, children born preterm (< 37 weeks) were associated with lower BMI, reflecting unique growth and developmental challenges [73].

 Although the average daily smart device use in this study was less than an hour, prior research indicated that even similar amounts of tablet use may be associated with adverse outcomes in young children [8]. Overall, our findings highlight the importance of considering emerging technologies, such as smart device use, in relation to BMI within the broader family context, in order to better inform approaches for reducing early health risks and promoting healthy behaviors.

This study has several important implications. First, our findings underscore the potential value of fostering high-quality parent-child interactions to mitigate possible adverse effects of smart device use and support healthy behaviors in preschoolers [2, 74]. Activities such as talking, listening, reading, teaching, and providing verbal responses, elements assessed in this study, may be particularly relevant in this regard. Second, the sex-specific associations observed in this study, particularly among boys, suggest that early monitoring of screen behaviors may be especially salient for certain subgroups. Third, broader family lifestyle and environmental characteristics (e.g., parental BMI) were associated with children’s smart device use and BMI, indicating that the home environment may contribute to early behavioral patterns and related health risks [47, 48]. Early identification of these family characteristics could help guide targeted support for promoting healthier routines within families. Finally, in the current digital era, smart devices have largely replaced traditional screens and become nearly unavoidable in young children’s daily lives [25]. Existing tools, such as the American Academy of Pediatrics’ Family Media Use Plan, can possibly assist families in establishing structured and developmentally appropriate media routines [2].

### Strengths and limitations

The strengths of the present study include its investigation of the bidirectional relationship between smart device use and BMI in young children, offering valuable insights into developmental patterns. This analysis was further strengthened by the study’s longitudinal design. Focusing on preschoolers provided a unique perspective on this critical period of rapid growth and habit formation. In addition, this study examined the complex interplay of this relationship by incorporating mother-child interactions and child sex as moderators. The inclusion of both maternal and paternal variables, such as BMI and educational levels, further enhanced our understanding of family dynamics. 

This study has several limitations. First, the recruitment of participants from metropolitan Taipei, primarily women of advanced age and higher socioeconomic status, may have limited the generalizability of the findings. In addition, the inclusion of fathers strengthened the study findings by incorporating paternal influences and family-level dynamics in child development. Nevertheless, requiring fathers’ participation in this study may have introduced selection bias, as families willing or able to involve both parents may have been more likely to represent relatively favorable marital and family relationships. Second, the use of self-reported questionnaires may have introduced biases because mothers might not have accurately recalled events or may have hesitated to report problematic interactions [75]. Although parental reporting remains the predominant method for assessing young children’s screen time [38], future studies should utilize applications that automatically record real-world device usage to provide more objective usage data [76]. Additionally, children’s height and weight were reported by mothers, based on the *Children’s Health Handbook*, which may have introduced reporting bias compared with direct anthropometric assessments by researchers. Third, in this study, smart device use was examined solely as time spent on smartphones and tablets. Other screen-based behaviors or contextual factors (e.g., timing, co-use, or total screen exposure) may covary with smart device use and therefore warrant consideration in future research. Fourth, the content of the media consumed was not examined in this study. Future research should address this gap by exploring how different types of media, such as educational or gaming apps, affect development across social, cognitive, and physical domains. Finally, although plausible biological and behavioral pathways may link smart device use with child BMI or vice versa, our study did not directly measure potential mediators such as circadian rhythm disruption, dietary patterns, physical activity, or self-regulation. Future research should include objective assessments of these factors to clarify biological plausibility and test their mediating roles.

## Conclusions

In this study, we identified bidirectional associations between increased smart device use and increased BMI, which in turn elevated smart device use among preschoolers, particularly among boys and children with a low level of mother-child interactions. High-quality mother-child interactions were found to mitigate this association. Taken together, these findings underscore the importance of fostering strong parent-child relationships and supportive family environments in promoting healthy behaviors and overall development. Future studies should analyze media content and children’s self-regulation to gain a more comprehensive understanding of how these factors affect the relationship between smart device use and BMI.

## Supplementary Information

Additional file 1: Appendix. Construction of the basic RI-CLPM. Supplemental Table S1. Comparison of baseline characteristics between nonparticipants with missing data and participants. Supplemental Table S2. Directional associations between children’s smart device use and BMI evaluated using the basic RI-CLPM. Supplemental Table S3. Model fit comparison for moderation effects in RI-CLPM of children’s smart device use and BMI.

Additional file 2: STROBE checklist

## Data Availability

Requesting data access for this project requires contact with a corresponding author. Data release requires permission from MacKay Memorial Hospital’s Institutional Review Board and adherence to the terms of the research cooperation agreement. Funding organizations and our ethics committee specified these requirements.

## Abbreviations

BMI: Body mass index
RI-CLPM: Random-intercept cross-lagged panel model
CI: Confidence interval
CFI: Comparative fit index
LEAPP-HIT: Longitudinal Examination Across Prenatal and Postpartum Health in Taiwan
RMSEA: Root mean square error of approximation
SRMR: Standardized root mean square residual
SD: Standard deviation
NTD: New Taiwan dollar
