# Supplementary material for: Bidirectional associations between smart device use and body mass index among children aged 3 to 5 years: a longitudinal study
Source: Int J Behav Nutr Phys Act. 2026 Feb 4;23:18. doi: 10.1186/s12966-026-01883-3 (PMC12958733; doi:10.1186/s12966-026-01883-3)
Supplement: Supplementary file 1 — Additional file 1: Appendix. Construction of the basic RI-CLPM. Supplemental Table S1. Comparison of baseline characteristics between nonparticipants with missing data and participants. Supplemental Table S2. Directional associations between children’s smart device use and BMI evaluated using the basic RI-CLPM. Supplemental Table S3. Model fit comparison for moderation effects in RI-CLPM of children’s smart device use and BMI. [file 12966_2026_1883_MOESM1_ESM.docx]

**Appendix: Construction of the basic RI-CLPM**

**Step 1: Specification of within-person components**

The observed variables for smart device use and BMI *z*-scores at the ages of 3, 4, and 5 years were modeled to define within-person components. The structural relationships for these components included the following:

- Cross-lagged paths: These paths capture the directional and temporal associations between smart device use and BMI *z*-scores at adjacent time points, demonstrating how changes in one variable affect the other (eg, device use at the age of 4 years → BMI at the age of 5 years).
- Autoregressive paths: These paths represent the stability of smart device use and BMI *z*-scores within individuals across consecutive time points, reflecting the carry-over effects of deviations from the mean (eg, device use at the age of 4 years → device use at the age of 5 years).

**Step 2: Incorporation of between-person components**

Latent variables were introduced to capture stable, time-invariant individual differences in the observed variables:

- Random intercept for smart device use (B-Device): This variable accounts for stable, between-person differences in smart device use at all time points.
- Random intercept for BMI *z*-scores (B-BMI): This variable captures stable, between-person differences in BMI *z*-scores at all time points.

Random intercepts were specified with factor loadings fixed at 1 for all time points to separate stable individual differences from dynamic changes over time. Significant variances in both intercepts confirmed the presence of stable, trait-like differences in smart device use and BMI *z*-scores.

**Step 3: Specification of covariances**

Covariances were included to account for associations within and between components:

- Between-person covariance: This covariance captures the stable association between smart device use and BMI *z*-scores through random intercepts.
- Within-person covariance: This covariance accounts for shared, time-specific effects by allowing the residual variances of within-person components to covary at each time point.

**Supplemental Table S1. Comparison of baseline characteristics between nonparticipants with missing data and participants**

| **Variable** | **Included**  **(*n* = 590)** | **Excluded**  **(*n* = 276)** | ***p* value** |
| --- | --- | --- | --- |
| Maternal age, n (%) |  |  |  |
| < 35 | 361 (61.82) | 188 (68.12) | .068 |
| ≥ 35 | 223 (38.18) | 88 (31.88) |  |
| Parental educational level, n (%) |  |  |  |
| Both with a graduate school degree or higher | 324 (55.29) | 175 (63.41) | .078 |
| Either with a graduate school degree or higher | 166 (28.33) | 65 (23.55) |  |
| Both with a college degree or lower | 96 (16.38) | 36 (13.04) |  |
| Family monthly income,^a^ n (%) |  |  |  |
| ≤ NTD100 000 | 347 (59.62) | 161 (58.33) | .720 |
| > NTD100 000 | 235 (40.38) | 115 (41.67) |  |
| Parity, n (%) |  |  |  |
| Primiparous | 355 (60.17) | 181 (65.58) | .127 |
| Multiparous | 235 (39.83) | 95 (34.42) |  |
| Gestational age (in weeks), n (%) |  |  |  |
| <37 | 45 (7.64) | 13 (4.76) | .117 |
| ≥37 | 544 (92.36) | 260 (95.24) |  |
| Child sex, n (%) |  |  |  |
| Boy | 299 (50.68) | 139 (50.55) | .971 |
| Girl | 291 (49.32) | 136 (49.45) |  |

Abbreviations: NTD, New Taiwan dollar.

^a^Average exchange rate in 2023: US$1.00 = NT$30.03.

**Supplemental Table S2. Directional associations between children’s smart device use and BMI evaluated using the basic RI-CLPM**

| **Association** | **Standardized estimate (95% CI)** |
| --- | --- |
| Cross-lagged effects |  |
| *Ages 3 to 4* |  |
| Smart device use at age 3 → BMI at age 4 | 0.23 (−0.04, 0.50) |
| BMI at age 3 → Smart device use at age 4 | −0.01 (−0.28, 0.27) |
| *Ages 4 to 5* |  |
| Smart device use at age 4 → BMI at age 5 | 0.17 (−0.17, 0.52) |
| BMI at age 4 → Smart device use at age 5 | **0.36 (0.05, 0.67)** |
| Autoregressive effects |  |
| *Ages 3 to 4* |  |
| Smart device use at age 3 → Smart device use at age 4 | −0.30 (−0.87, 0.27) |
| BMI at age 3 → BMI at age 4 | 0.09 (−0.25, 0.43) |
| *Ages 4 to 5* |  |
| Smart device use at age 4 → Smart device use at age 5 | 0.43 (−0.20, 1.06) |
| BMI at age 4 → BMI at age 5 | 0.05 (−0.33, 0.43) |
| Within-person covariances |  |
| Smart device use at age 3 ↔ BMI at age 3 | −0.09 (−0.36, 0.19) |
| Smart device use at age 4 ↔ BMI at age 4 | 0.06 (−0.25, 0.37) |
| Smart device use at age 5 ↔ BMI at age 5 | −0.07 (−0.32, 0.18) |
| Between-person covariance |  |
| B-Smart device ↔ B-BMI | 0.12 (−0.03, 0.28) |
| Variances |  |
| σ_Smart device_^2^ | **0.39 (0.18, 0.60)** |
| σ_BMI_^2^ | **0.67 (0.46, 0.88)** |
| Fit indices |  |
| χ^2^ (df), *p* value | 0.01(1), 0.921 |
| RMSEA | 0.00 |
| CFI | 1.00 |
| SRMR | 0.00 |

Abbreviations: RI-CLPM, random-intercept cross-lagged panel model; BMI, body mass index; RMSEA, root mean square error of approximation; CFI, Comparative fit index; SRMR, standardized root mean square residual

**Bold values** indicate standardized estimates where 95% CIs do not include zero.

**Supplemental Table S3. Model fit comparison for moderation effects in RI-CLPM of children’s smart device use and BMI**

| **Directional association** | **RMSEA** | **SRMR** | **CFI** | **χ^2^** | **df** | ***p* value** | **Δχ^2^** | **df** | ***p* value** |
| --- | --- | --- | --- | --- | --- | --- | --- | --- | --- |
| Child sex |  |  |  |  |  |  |  |  |  |
| Unconstrained | 0.05 | 0.02 | 0.99 | 3.54 | 2 | .171 | — | — | — |
| Constrained | 0.10 | 0.08 | 0.84 | 58.17 | 14 | <.001 | 53.53 | 12 | <.001 |
| Mother-child interaction |  |  |  |  |  |  |  |  |  |
| Unconstrained | 0.00 | 0.02 | 1.00 | 1.19 | 2 | .550 | — | — | — |
| Constrained | 0.08 | 0.08 | 0.88 | 38.42 | 14 | <.001 | 38.07 | 12 | <.001 |

Abbreviations: RI-CLPM, random-intercept cross-lagged panel model; BMI, body mass index; RMSEA, root mean square error of approximation; SRMR, standardized root mean square residual; CFI, comparative fit index.
