# Supplementary material for: Bidirectional associations between smart device use and body mass index among children aged 3 to 5 years: a longitudinal study
Source: Int J Behav Nutr Phys Act. 2026 Feb 4;23:18. doi: 10.1186/s12966-026-01883-3 (PMC12958733; doi:10.1186/s12966-026-01883-3)
Supplement: Supplementary file 2 — Additional file 2: STROBE checklist [file 12966_2026_1883_MOESM2_ESM.docx]

**STROBE Statement—checklist of items that should be included in reports of observational studies**

**Title**: Bidirectional associations between smart device use and body mass index among children aged 3 to 5 years: a longitudinal study

|  | Item No. | Recommendation | Page  No. | Relevant text from manuscript |
| --- | --- | --- | --- | --- |
| **Title and abstract** | 1 | (*a*) Indicate the study’s design with a commonly used term in the title or the abstract | 1 | Bidirectional associations between smart device use and body mass index among children aged 3 to 5 years: a longitudinal study |
|  |  | (*b*) Provide in the abstract an informative and balanced summary of what was done and what was found | 3-4 | From the manuscript, lines: 45-77 |
| Introduction | | | |  |
| Background/rationale | 2 | Explain the scientific background and rationale for the investigation being reported | 6-7 | From the manuscript, lines: 130-146 |
| Objectives | 3 | State specific objectives, including any prespecified hypotheses | 6-7 | From the manuscript, lines: 136-146 |
| Methods | | | |  |
| Study design | 4 | Present key elements of study design early in the paper | 7 | From the manuscript, at the beginning of the method section. “Study Design and Data Collection” |
| Setting | 5 | Describe the setting, locations, and relevant dates, including periods of recruitment, exposure, follow-up, and data collection | 7 | From the manuscript, at the beginning of the method section. “Study design and data collection” |
| Participants | 6 | (*a*) *Cohort study*—Give the eligibility criteria, and the sources and methods of selection of participants. Describe methods of follow-up  *Case-control study*—Give the eligibility criteria, and the sources and methods of case ascertainment and control selection. Give the rationale for the choice of cases and controls  *Cross-sectional study*—Give the eligibility criteria, and the sources and methods of selection of participants | 7-8 | Methods section, under “Sample”, we stated eligibility criteria; *“We included Mandarin-speaking women aged ≥ 20 years who were in early pregnancy (< 16 weeks), intended to carry to full term, and had partners willing to participate. We specifically examined data on smart device use and BMI collected during the 3-, 4-, and 5-year postpartum follow-up assessments.”*  *-*Page 7: Source population and recruitment setting, “Parents and children were recruited from the Longitudinal Examination Across Prenatal and Postpartum Health in Taiwan (LEAPP-HIT), an ongoing prospective study initiated in 2011 in metropolitan Taipei.”  - Page 7: follow-up method and missing data described how the study followed up and handled with missing data. |
|  |  | (*b*) *Cohort study*—For matched studies, give matching criteria and number of exposed and unexposed  *Case-control study*—For matched studies, give matching criteria and the number of controls per case |  | - |
| Variables | 7 | Clearly define all outcomes, exposures, predictors, potential confounders, and effect modifiers. Give diagnostic criteria, if applicable | 8-10 | From the manuscript, under “Measure”, lines 174-226. We present all the variables for assessment in this study. |
| Data sources/ measurement | 8* | For each variable of interest, give sources of data and details of methods of assessment (measurement). Describe comparability of assessment methods if there is more than one group | 8-10 | From the manuscript,  - **Smart device use**: Reported by mothers using validated questions about typical weekday/weekend usage. The weighted average was calculated, which relied on previous study measurements. (p.8, lines 178-190)  - **BMI z-scores**: Derived from mother-reported height/weight recorded in children’s health handbooks maintained by health professionals. Standardized against WHO/Taiwan growth charts. (p.9, lines 191-197)  - **Mother-child interaction**: Assessed using Brigance Parent-Child Interactions Scale. Converted to binary (high vs. low) based on quartile cut-off. (p.9, lines 199-207)  - **Child sex**: Documented by health care providers at birth. (p.9, lines 208-210)  - **Covariates:** Collected via baseline surveys and validated instruments, including self-reported parental BMI, maternal depression (EPDS), and outdoor activity. (p.9-10, lines 211-226)  All measurements were applied uniformly across groups and time points. |
| Bias | 9 | Describe any efforts to address potential sources of bias | 10-11 | In this study, the RI-CLPM was used to analyse.  - Used full-information maximum likelihood (FIML) to address missing data, supported by Little’s MCAR test  - Applied robust standard errors for nonnormality  - Used RI-CLPM to account for time-invariant confounding, reducing bias from stable individual/family traits (p.10, and supplementary appendix)  - Acknowledged possible recall and reporting bias in maternal-reported screen time and interaction, and suggested improvements (p.22, lines 477-484). |
| Study size | 10 | Explain how the study size was arrived at | 7-8 | From the manuscript, under the “Sample”, explained how final sample size was included. The study included 866 eligible participants from a longitudinal cohort. The analytic sample (n = 590) was derived based on participants with concurrent BMI and smart device use data at any of the 3-, 4-, or 5-year waves. |

Continued on next page

| Quantitative variables | 11 | Explain how quantitative variables were handled in the analyses. If applicable, describe which groupings were chosen and why | 8-10 | Smart device use and BMI z-scores were analyzed as continuous variables across three-time points. Mother-child interaction scores were dichotomized into “high” (top 25%) and “low” (lower 75%) to test moderation. Several covariates were categorized for interpretability, including maternal age (≥35 years), gestational age (term vs. preterm), and maternal depression (EPDS ≥13). |
| --- | --- | --- | --- | --- |
| Statistical methods | 12 | (*a*) Describe all statistical methods, including those used to control for confounding | 10-11 | We described the concept of RI-CLPM under the “Statistical Analyses”, and also on supplementary appendix. Random-Intercept Cross-Lagged Panel Modeling (RI-CLPM) was used to account for time-invariant confounders by separating between- and within-person variance. Covariates were regressed onto between-person components. Robust standard errors and maximum likelihood estimation were applied. |
|  |  | (*b*) Describe any methods used to examine subgroups and interactions | 11 | Multiple-group RI-CLPMs were conducted for mother–child interaction and child sex. Chi-square difference tests compared constrained vs. unconstrained models to assess moderation. |
|  |  | (*c*) Explain how missing data were addressed | 11 | Full-information maximum likelihood (FIML) was used to address missing data. Little’s MCAR test confirmed that data were missing completely at random (χ² = 175.26, P = .70). |
|  |  | (*d*) *Cohort study*—If applicable, explain how loss to follow-up was addressed  *Case-control study*—If applicable, explain how the matching of cases and controls was addressed  *Cross-sectional study*—If applicable, describe analytical methods taking account of the sampling strategy | 7-8 | Under the sub-section of “Sample” was explained how the study presented the loss to follow-up. In addition, we examined a comparison of included vs. excluded participants provided in Supplemental Table 2. |
|  |  | (*e*) Describe any sensitivity analyses |  | No sensitivity analyses were conducted. |
| Results | | | | |
| Participants | 13* | (a) Report numbers of individuals at each stage of study—eg numbers potentially eligible, examined for eligibility, confirmed eligible, included in the study, completing follow-up, and analyzed | 7,8/12 | Under the “Descriptive statistics” we summarized the descriptive statistics of the participants. In addition, the “Sample” has been described in the method section. The initial eligible cohort consisted of 866 participants with singleton births after December 2016, when smart device use data collection began. Among them, 590 children had valid BMI z-scores and smart device use data for at least one time point (age 3, 4, or 5 years) and were included in the final analytic sample. A detailed summary of participant characteristics is provided in the main text and Table 1. |
|  |  | (b) Give reasons for non-participation at each stage | 7-8 | 276 participants were excluded due to missing data on both smart device use and BMI z-scores at all waves, making them ineligible for inclusion in the longitudinal model. These missing data were due to incomplete follow-up or questionnaire nonresponse. A comparison of included and excluded participants is presented in Supplemental Table 1. |
|  |  | (c) Consider use of a flow diagram |  | Not included, but sample comparison table is provided in the supplement. |
| Descriptive data | 14* | (a) Give characteristics of study participants (eg demographic, clinical, social) and information on exposures and potential confounders | 12, and Table 1 | Participant characteristics—including demographics, maternal age, parental BMI, education, income, maternal depression, gestational age, child sex, and outdoor activity—are summarized in the Results section (p.12, lines 264-272) and fully detailed in Table 1. |
|  |  | (b) Indicate number of participants with missing data for each variable of interest | Table 1 | Missing data patterns are not fully described in Table 1, however we followed the reviewer comment in this manuscript revision. In addition we clarify with the Little test in our statistica analyses. |
|  |  | (c) *Cohort study*—Summarise follow-up time (eg, average and total amount) |  | The study followed participants at three time points—when children were aged 3, 4, and 5 years—over a 3-year follow-up period. |
| Outcome data | 15* | *Cohort study*—Report numbers of outcome events or summary measures over time | 12-17, and Table 1. | Mean values and standard deviations for repeated measures of variables were reported in Table 1. Figures 1–3 and Tables 2–4 detail cross-lagged associations across time points. |
|  |  | *Case-control study—*Report numbers in each exposure category, or summary measures of exposure |  |  |
|  |  | *Cross-sectional study—*Report numbers of outcome events or summary measures |  |  |
| Main results | 16 | (*a*) Give unadjusted estimates and, if applicable, confounder-adjusted estimates and their precision (eg, 95% confidence interval). Make clear which confounders were adjusted for and why they were included |  | Standardized estimates (β) with 95% confidence intervals are reported for all cross-lagged and autoregressive paths in Figures 1–3 and the Results section (p.12–17). Covariates were included based on theoretical and empirical relevance and were regressed onto between-person components in the RI-CLPM (see Table 4). The RI-CLPM structure controls for time-invariant confounding at both individual and family levels. |
|  |  | (*b*) Report category boundaries when continuous variables were categorized |  | **Mother-child interaction** was dichotomized based on the upper quartile (≥75th percentile) to define the “high” group |
|  |  | (*c*) If relevant, consider translating estimates of relative risk into absolute risk for a meaningful time period |  |  |

Continued on next page

| Other analyses | 17 | Report other analyses done—eg analyses of subgroups and interactions, and sensitivity analyses |  |  |
| --- | --- | --- | --- | --- |
| Discussion | | | | |
| Key results | 18 | Summarise key results with reference to study objectives | 17-18 | The Discussion (p.17, lines 355-363) begins with a clear summary of the key findings, directly referencing the study objectives. It emphasizes the bidirectional association between smart device use and BMI among preschoolers. It highlights that boys and children with low-quality mother-child interaction were particularly vulnerable to these reciprocal effects. |
| Limitations | 19 | Discuss limitations of the study, taking into account sources of potential bias or imprecision. Discuss both direction and magnitude of any potential bias | 22-23 | Study limitations were discussed in detail on p.22-23 (lines 470-494). These include the limited generalizability due to sampling from metropolitan Taipei, potential biases from self-reported measures, and the lack of media content analysis. The authors acknowledge that recall or reporting bias may exist and note that missingness, while handled using FIML and tested with Little’s MCAR test, could still influence estimates. The Discussion reflects on the direction and magnitude of possible biases. |
| Interpretation | 20 | Give a cautious overall interpretation of results considering objectives, limitations, multiplicity of analyses, results from similar studies, and other relevant evidence | 17-23 | The overall interpretation, we carefully integrated the study’s aims, observed results, and prior literature. We avoid overstating conclusions, instead noting cautious interpretations based on observational data. The authors reflect on the broader evidence base linking media use and obesity, clarify the unique contribution of their RI-CLPM approach, and call for further research on mechanisms such as media content and self-regulation. |
| Generalisability | 21 | Discuss the generalisability (external validity) of the study results | 22 | Generalisability is addressed in the Limitations p.22 (lines 470-472). , noting that the urban Taiwanese sample with relatively high SES may not reflect other populations. However, the developmental processes explored are likely relevant cross-culturally, and the findings may apply to other urbanized, tech-saturated settings. |
| Other information | |  | | |
| Funding | 22 | Give the source of funding and the role of the funders for the present study and, if applicable, for the original study on which the present article is based | 24-25 | Funding information is provided under the Declarations section. The study was funded by the Ministry of Science and Technology in Taiwan. The funders had no role in the study design, data collection, analysis, interpretation, or manuscript writing. |

*Give information separately for cases and controls in case-control studies and, if applicable, for exposed and unexposed groups in cohort and cross-sectional studies.

**Note:** An Explanation and Elaboration article discusses each checklist item and gives methodological background and published examples of transparent reporting. The STROBE checklist is best used in conjunction with this article (freely available on the Web sites of PLoS Medicine at http://www.plosmedicine.org/, Annals of Internal Medicine at http://www.annals.org/, and Epidemiology at http://www.epidem.com/). Information on the STROBE Initiative is available at www.strobe-statement.org.
